# A critical discussion of the current availability of lithium and zinc for use in batteries

**DOI:** 10.1038/s41467-024-48368-0

**Published:** 2024-05-14

**Authors:** Alessandro Innocenti, Dominic Bresser, Jürgen Garche, Stefano Passerini

**Affiliations:** 1 Helmholtz Institute Ulm (HIU), Helmholtzstrasse 11, 89081 Ulm, Germany; 2 Karlsruhe Institute of Technology (KIT), P.O. Box 3640, 76021 Karlsruhe, Germany; 3 Institute for Theoretical Chemistry, University of Ulm, Oberberghof 7, 89081 Ulm, Germany; 4 Department of Chemistry, Sapienza University of Rome, Piazzale A. Moro 5, 00185 Rome, Italy; 5 Present Address: Zentrum für Sonnenenergie- und Wasserstoff-Forschung Baden-Württemberg, 89081 Ulm, Germany

**Keywords:** Batteries, Batteries

## Abstract

Aqueous zinc batteries are currently being explored as potential alternatives to non-aqueous lithium-ion batteries. In this comment, the authors highlight zinc’s global supply chain resilience and lower material costs yet caution about its higher mass requirement for comparable charge storage.

## Teaser

In the literature on zinc-based batteries, it is often highlighted that zinc offers significant advantages over lithium due to its abundance, affordability, and accessibility. Additionally, aqueous rechargeable zinc batteries are promoted as a sustainable and cost-effective alternative to lithium-ion batteries, especially for renewable energy storage. The aim of this Comment is to provide a perspective on these statements, elucidating their foundations and implications and giving a quick but comprehensive background to authors and readers that deal with this topic, focusing specifically on batteries with zinc ions shuttling reversibly between the metallic negative electrode and the insertion-type positive electrode.

## Zinc and lithium compared: a matter of scale

**Fig. 1:**
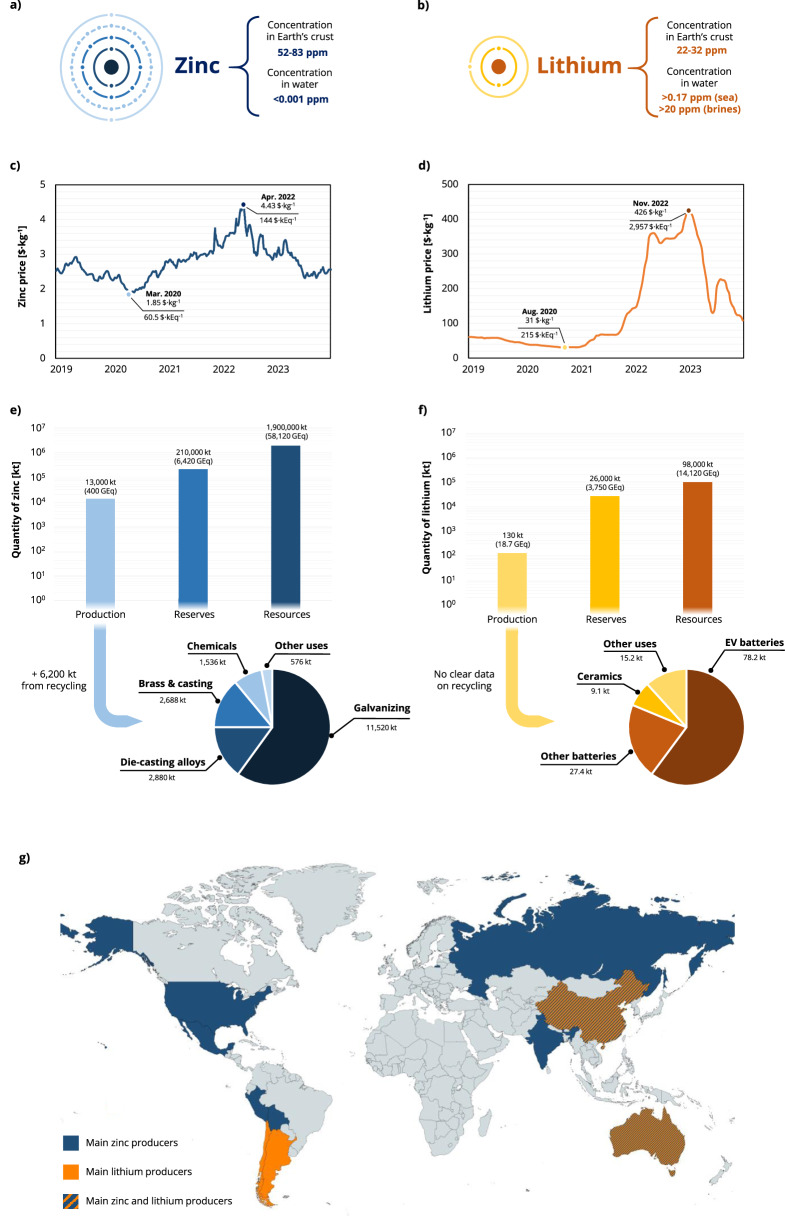
Overview of zinc and lithium cost, production, and distribution. Concentration in the Earth’s crust and in water of **a** zinc and **b** lithium. Trend of the price in the last 5 years (Nov. 2019–Nov. 2023) of **c** high-grade zinc metal and **d** battery-grade lithium metal (extrapolated from the lithium carbonate price). Production, reserves, and resources according to the United States Geological Survey (USGS) of **e** zinc and **f** lithium, together with a breakdown of the end uses and an indication of the amount of metal provided by recycling (end of 2022). (EV: electric vehicles). **g** Indication of the main zinc and lithium producing regions (created with MapChart).

The abundance of the two elements in the Earth’s crust is relatively similar: 52–83 ppm for zinc (Fig. 1a) and 22–32 ppm for lithium (Fig. 1b)^1^. In fact, a considerable amount of lithium is also found dissolved in seawater (0.17–0.19 ppm) and in continental brines (20–1500 ppm)^2^, while zinc is normally only present as a trace in water (<0.001 ppm), except in freshwaters or coastal waters contaminated by industrial discharges^3^. The cost of Zn and Li compounds used in the corresponding battery manufacturing, however, is rather different (Fig. 1c, d). In the last 5 years, the price of 99.95%-pure zinc metal oscillated between 1.85 and 4.4 $·kg^−1^, while battery-grade (99.5%) lithium carbonate used for lithium-ion battery (LIB) manufacturing lay between ~5.8 and 80 $·kg^−1^^4^. Rescaling the lithium carbonate price considering the lithium content (18.78 wt.%), we obtain a 31–426 $·kg^−1^ range for lithium. Considering the price per equivalent (i.e., the moles of electrons exchanged for every mole of element), we obtain instead 60.5–144 $·kEq^−1^ for zinc and 215–2957 $·kEq^−1^ for lithium. This measurement unit is valuable for comparing the metals based on the quantity required to store an equivalent amount of charge. The cost difference narrows because the zinc atom, despite being characterized by a two-electron reduction process, is 9.42 times heavier than the lithium one.

The main reason for this price discrepancy lies in the production scales and supply/demand dynamics (Fig. 1e, f). The current production of zinc amounts to 13,000 kt per year, with proven reserves of 210,000 kt, making zinc the fourth most extracted metal due to its widespread use in the galvanization of steel, alloys, and chemicals. Eight countries, namely China, Peru, Australia, India, the United States, Mexico, Bolivia, and Russia (listed in the order of decreasing production), are responsible for 77.2% of the global zinc production and own 78.5% of the reserves, and the identified global resources are ~1,900,000 kt, which have been constant since 1998^5^. In comparison, the extraction of lithium is nowadays only 130 kt per year and the reserves total 26,000 kt, which is two orders of magnitude less than the zinc volumes. 96.3% of the production comes from only four countries—Australia, Chile, China, and Argentina—in descending order of output, and these countries also control 77.7% of the known reserves. The global lithium resources amount currently to 98,000 kt, and they saw a substantial increase in this and the previous decade due to the high interest in this strategic metal (+1680% compared to 2013)^6^. Again, considering the production, reserves, and resources in the abovementioned equivalents instead of mass, the difference between zinc and lithium narrows significantly: the production becomes only one order of magnitude larger for the former, and reserves and resources are comparable, as these quantities are divided by the mass of material required to exchange a mole of electrons (32.69 kg·kEq^−1^ for zinc, 6.94 kg·kEq^−1^ for lithium). This highlights the significantly higher mass of zinc that would be required for energy storage applications to achieve the same charge capacity as lithium.

The consumption and production of lithium experienced strong growth in the last years because of its use in LIBs for consumer electronics, energy storage, and electric vehicles. In 2021 and 2022, the production of lithium was slightly lower than the demand, which caused a sharp price increase^7^, but recently the lithium prices decreased again due to a more relaxed battery demand in 2023 that created an oversupply situation^8^. The supply/demand balance of zinc is instead more stable thanks to the maturity of the underlying industry. Nevertheless, the estimated global stocks of zinc have been recently oscillating, a situation that reflected in its price, and in 2022 the element was added to the Critical Raw Materials list of the US Government^5^.

The current understanding is that the future demand for zinc (considering the forecasted growth of its present applications) can be easily met by the current extraction rate and the available reserves, and the widely distributed resources render the zinc supply less affected by geopolitical instabilities. Zinc also has a developed recycling industry that is able to provide almost one third of the global zinc demand, further strengthening its supply chain^9^. The case of lithium is instead more uncertain due to the rapidly changing situation concerning the available resources and the growth of the clean energy sector, as well as the current geographically confined lithium production and refinement^10^. Accordingly, an efficient recycling seems to be a fundamental step to achieve a reliable supply, but the LIB recycling industry is still in its infancy^11^.

## From raw materials to batteries

**Fig. 2:**
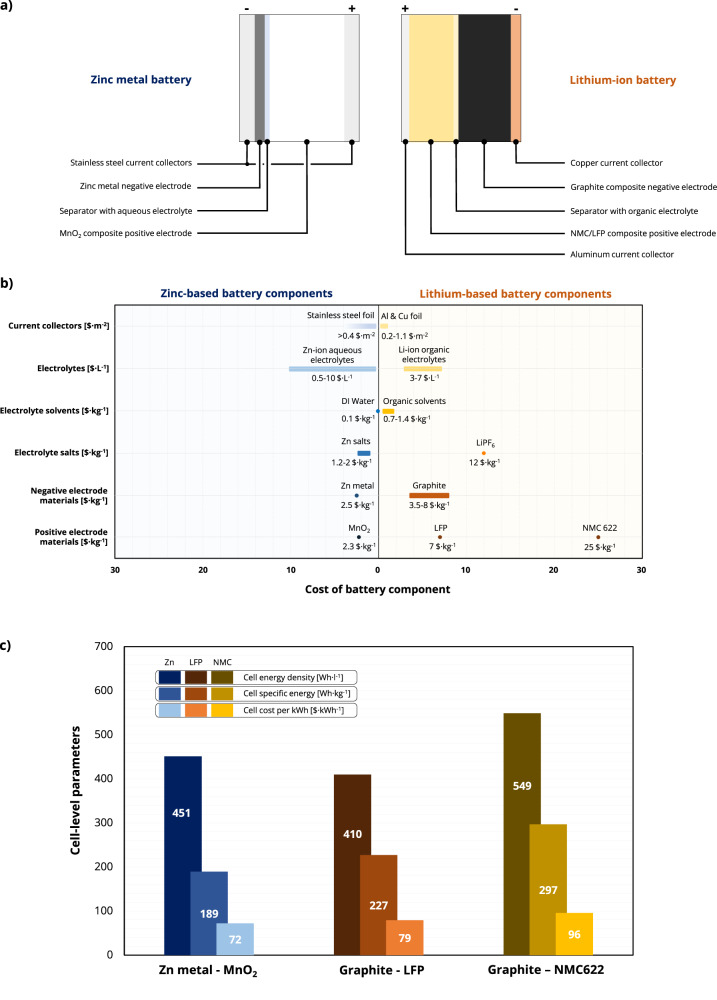
Cost analysis of zinc and lithium-ion-based batteries. **a** Schematic depiction of a rechargeable zinc battery and lithium-ion battery. **b** Comparison of the cost of the battery components of the two battery systems (zinc on the left, lithium-ion on the right) as of the end of 2023. **c** Results of the cost and energy density analysis of a small pack for home energy storage on a zinc-based battery and two different commercial lithium-ion batteries.

From the electrochemical point of view, the Li electrode has an equilibrium potential of −3.04 V vs. the standard hydrogen electrode (SHE), enabling the realization of rather high voltage cells (e.g., >2 V) based on the Li^+^ shuttling mechanism. On the other hand, a Zn metallic electrode has an equilibrium potential of −0.76 V vs. SHE, with a theoretical capacity of 820 mAh g^−1^ and 5855 mAh cm^−3^. Both ions (Li^+^, Zn^2+^) can be reversibly de/inserted in suitable positive electrode materials, making it possible to build rechargeable batteries based on this mechanism. Figure 2a depicts the structure of the aqueous zinc battery and non-aqueous LIBs that we analyse in this Comment. We focused our analysis only on zinc batteries based on mildly acidic aqueous electrolytes, due to the similar working principle with LIBs^12^. Nevertheless, the cell chemistry differs significantly, especially from a cost point of view. The current prices of the batteries’ components discussed in the following are reported in Fig. 2b.

The voltage window in aqueous zinc batteries is constrained by the water-based electrolytes: the thermodynamic electrochemical stability window of water would limit the theoretical maximum battery voltage to ~1.2 V^13^. The relatively high hydrogen evolution overvoltage of Zn and the use of (super)concentrated aqueous electrolytes can push this value and increase the practical operating voltage range improving then the cycle life by hindering water decomposition^14,15^. However, the higher cost and weight and the lower ionic conductivity of these concentrated electrolytes must be considered when evaluating the overall battery performance. The proposed salts vary from the cheap and widely available zinc sulfate (ZnSO_4_), chloride (ZnCl_2_), or acetate (Zn(CH_3_CO_2_)_2_ or ZnAc) to specialty chemicals as zinc triflate (ZnOTf_2_) or zinc di-bis(trifluoromethanesulfonyl)imide (ZnTFSI_2_)^16^. Currently, the last two salts can be practically employed only as additives, given their out-of-scale cost (>1000 $·kg^−1^). Zinc chloride is the cheapest and most soluble salt among all the cited ones, and a 2 m solution may cost as little as 0.5 $·L^−1^, while a superconcentrated 30 m electrolyte could reach approximately 10 $·L^−1^ (see Section 1 and Table S1 of the Supplementary Information for details on the calculations). For comparison, common non-aqueous electrolytes for LIBs, based on ca. 1 M solutions of lithium hexafluorophosphate (LiPF_6_) in solvents like ethylene carbonate and/or dimethyl carbonate, are currently priced 3–7 $·L^−1^ ^17^. Although the electrochemical stability window of such electrolytes is only approx. 3.5 V (from 1 to 4.5 V vs. Li^+^/Li), cell voltages >4 V are easily achieved due to stable passivation layers forming on both the anode (solid electrolyte interphase) and the cathode (cathode electrolyte interphase).

The current collector material of choice for aqueous zinc batteries is stainless-steel, since aluminum and copper corrode during operation in water-based acidic electrolytes^18^. Stainless-steel, while having a price comparable to aluminum, exhibits a resistivity at room temperature ~40 times higher than copper and 25 times higher than aluminum^19^. Additionally, the ductility and malleability of stainless-steel are notably inferior to those of the two other metals, making it relatively harder to obtain µm-thick foils suitable for the use as electrode current collectors^20^. Nevertheless, stainless-steel foils with a thickness as low as 25 µm are readily available for purchase.

Of the proposed positive electrode active materials for rechargeable zinc batteries, manganese dioxide (MnO_2_) is by far the most studied and promising^21–24^ thanks to its rather high specific capacity (305 mAh·g^−1^ in theory for a one-electron reaction), good cyclability and low cost (approximately 2.3 $·kg^−1^). In fact, this oxide is already mass-produced for primary alkaline zinc batteries, and manganese is a cheap and widespread transition metal. The negative electrode is usually a zinc metal foil^25^. A typical aqueous Zn-MnO_2_ battery with a mildly acidic electrolyte has an average voltage of around 1.35 V, while commercial LIBs with positive electrode chemistries such as LiFePO_4_ (LFP) and Li[Ni_0.6_Mn_0.2_Co_0.2_]O_2_ (NMC622) versus a composite graphite negative electrode reach 3.2 and 3.7 V, respectively.

We simulated the production of a small battery pack for home electrochemical energy storage, used, for instance, to store energy generated via photovoltaic panels, assuming near ideal conditions for the battery: 300 mAh·g^−1^ practical capacity for the MnO_2_-based positive electrode, 100% utilization of the zinc metal negative electrode, a price of the zinc foil equal to the zinc metal price (no production overhead for the foil), minimum aqueous electrolyte cost of 0.5 $·L^−1^ (i.e., a 2 m ZnCl_2_ solution in deionized water), and 20-µm-thick stainless-steel current collectors at a cost of 0.8 $·m^−2^ (see the Section 2 of the Supplementary Information for the determination of the current collector cost). We also took care of removing the costs associated with the dry room and the negative electrode production from the used model (BatPaC 5.0)^26^. The comparison is made with standard LFP and NMC622 LIBs with the current commercial prices, as reported in Fig. 2b^26,27^. More details about these configurations and the results of the simulations are given in Section 3 and in Tables S2 and S3 of the Supplementary Information.

Figure 2c shows the results of this analysis: the Zn-MnO_2_ battery has the lowest cost among the systems at the cell level (72 $·kWh^−1^ vs. 79 $·kWh^−1^ for LFP and 96 $·kWh^−1^ for NMC622) thanks to the price competitiveness of its components. Nevertheless, it also has the lowest specific energy (189 Wh·kg^−1^ vs 227 Wh·kg^−1^ for LFP and 297 Wh·kg^−1^ for NMC), mainly due to the low cell voltage achievable for the given aqueous cell chemistry. The volumetric energy density is higher than the one of LFP, thanks to the high density of MnO_2_ (5.03 g·cm^−3^) and zinc (7.14 g·cm^−3^) compared to LFP (3.45 g·cm^−3^) and graphite (2.26 g·cm^−3^). Notably, this zinc-based battery pack contains ~13 kg of zinc metal, while the same energy is stored in the lithium-based battery packs with ~1 kg of lithium.

Overall, in these ideal conditions, the zinc battery may be cost-competitive with LFP and cheaper than NMC, but not drastically more economical. It is important to note, though, that the realization of stable and rechargeable Zn-MnO_2_ aqueous batteries with comparable performance as the two very well-established LIBs discussed herein has yet to be practically demonstrated. Moreover, the resources needed in terms of the amount of metal per Wh could lead, in general, to higher zinc costs, too.

## Outlook

In this comment, we assessed common assertions in rechargeable zinc battery literature, examining prevailing claims about zinc’s abundance and cost-effectiveness compared to lithium and, eventually, LIBs. From a raw material perspective, we underscore zinc’s supply chain resilience and its developed recycling industry. Nonetheless, we would like to stress the much higher mass of zinc required to store the same amount of charge compared to lithium, which may drive an increase in the zinc price in case of its widespread application in batteries. We also emphasize significant material cost differences which are currently in favor of the aqueous zinc rechargeable batteries. Despite this potential cost competitiveness under ideal conditions, however, it should be noted that there are no Zn-MnO_2_ aqueous batteries yet with the ideal performances assumed in this work, i.e., the same performance as provided by the two very well-established LIB chemistries used for comparison, especially for concerning the zinc negative electrode utilization and the stability of the aqueous electrolyte.

We limited our focus to the model of mildly acidic aqueous Zn-MnO_2_ rechargeable cell chemistry and did not analyse other zinc-based systems investigated in current research, such as rechargeable alkaline batteries^27,28^ or zinc-halogen batteries^29^, which are capable, at a lab-scale level, of improving the cell voltage compared to our model system. However, it should be pointed out that considerations about the zinc supply chain and the cost of the raw materials apply to these chemistries, too, and they could be the subject of similar assessments in future works.

### Supplementary information

Supplementary Information

## Data Availability

The BatPaC model used in this work is an open-source model that can be requested from the Argonne National Laboratory. All the hypotheses behind the simulations, the parameters used in the model, and the main results are reported in the Supplementary Information of this article.
