# Supplementary material for: A critical discussion of the current availability of lithium and zinc for use in batteries
Source: Nat Commun. 2024 May 14;15:4068. doi: 10.1038/s41467-024-48368-0 (PMC11094038; doi:10.1038/s41467-024-48368-0)
Supplement: Supplementary file 1 — Supplementary Information [file 41467_2024_48368_MOESM1_ESM.pdf]

A Critical Discussion of the Current Availability of Lithium and Zinc for Use in Batteries

*Alessandro Innocenti[1,2,†], Dominic Bresser[1,2], Jürgen Garche[3], Stefano Passerini[1,2,4 *]*

[1] Helmholtz Institute Ulm (HIU), Helmholtzstrasse 11, 89081 Ulm, Germany
[2] Karlsruhe Institute of Technology (KIT), P.O. Box 3640, 76021 Karlsruhe, Germany
[3] Institute for Theoretical Chemistry, University of Ulm, Oberberghof 7, 89081 Ulm, Germany
[4] Department of Chemistry, Sapienza University of Rome, Piazzale A. Moro 5, 00185 Rome, Italy

[†] Present address: Zentrum für Sonnenenergie- und Wasserstoff-Forschung Baden-Württemberg, 89081 Ulm, Germany

[*] Corresponding author: stefano.passerini@kit.edu

Supplementary Information

1. Electrolyte cost

The salts for the zinc-ion batteries mentioned in the text which are also bulk chemicals are zinc chloride ($ZnCl_2$), zinc sulfate ($ZnSO_4$) and zinc acetate (ZnAc). Their cost was obtained by averaging the values found in a website where they were offered in bulk quantities.[1–3] Since $ZnSO_4$ and ZnAc are usually sold respectively in the heptahydrate and dihydrate forms, their price was corrected take into account only the dry fraction. We calculated then the aqueous electrolytes density and cost, assuming a bulk price of deionized water (conductivity <0.5 $\mu S \cdot cm^{-1}$) of 0.1 $\$ \cdot kg^{-1}$ and considering both a 2 m solution and a solution with molality equal to the solubility limit of these salts in water at room temperature (see **Table S1**). We chose to perform the simulations with the cheapest electrolyte, i.e., the 2 m $ZnCl_2$ one, reducing further the price to 0.5 $\$ \cdot L^{-1}$.

Table S1 – Details on the zinc salts and the related aqueous electrolytes for zinc batteries

Zinc salt	Salt cost [$\$ \cdot kg^{-1}$]	Molecular weight [$g \cdot mol^{-1}$]	Electrolyte molality [$mol \cdot kg^{-1}$]	Electrolyte density [$g \cdot cm^{-3}$]	Electrolyte cost [$\$ \cdot L^{-1}$]
$ZnCl_2$	1.2	136.29	2<br>30	1.19 [a]<br>2.10 [a]	0.57<br>10.45
$ZnSO_4$	2.0	161.47	2<br>3.3	1.28 [a]<br>1.40 [a]	0.98<br>1.64
ZnAc	1.7	183.48	2<br>3.5	1.15 [b]<br>1.30 [b]	0.77<br>1.24

[a] Retrieved from Perry's Chemical Engineers' Handbook[4]; [b] Assumed density of the zinc acetate solutions, based on an interpolation between the zinc acetate and water densities

2. Stainless-steel foil cost

The cost of the stainless-steel foils was calculated with the formula

$$C_{SS} = (c_{SS} + c_p) \cdot \rho_{SS} \cdot t_{SS}$$

where C_{SS} is the current collector cost per square meter, c_{SS} is the stainless steel cost per kg (assumed to be 2 $\$\cdot kg^{-1}$, according to the latest bulk prices),[5] c_p is the processing cost to produce a foil per kg of material, ρ_{SS} is the stainless steel density (8 $g\cdot cm^{-3}$), and t_{SS} is the thickness of the stainless steel foil.

Currently, the processing costs to obtain 15 µm and 8 µm thick aluminium and copper foils are respectively 1.95 $\$\cdot kg^{-1}$ and 2.6 $\$\cdot kg^{-1}$,[6,7] but no information were found on the processing costs of steel foils. Since we expect that the processing of steel is more difficult due to the lower ductility and malleability than these other two metals, we assumed a cost of 3 $\$\cdot kg^{-1}$ for a 20 µm foil. Hence, according to the reported formula, we obtain a steel foil price of 0.8 $\$\cdot m^{-2}$. The value of 0.4 $\$\cdot m^{-2}$ reported in **Figure 2.b** of the main text was obtained by setting a stainless steel foil thickness of 10 µm and keeping the processing costs constant.

3. Simulations of battery cost and energy density

The assessment of energy density and cost involved the utilization of the open source BatPaC 5.0 software. A detailed explanation of the BatPaC model can be found in the corresponding report released by the Argonne National Laboratory.[8] This model is specifically designed for simulating battery packs with specified energy and power ratings, taking into account the costs associated with various components such as active materials, conductive carbon, binders, separators, electrolytes, current collectors, casings, pack current collectors, cooling systems, labor, and overheads. Additionally, it factors in the investment costs related to the production site.

The simulated battery pack is intended for domestic energy storage, featuring a power rating of 7 kW and an energy rating of 11.5 kWh. It consists of 36 cells in each module, with a total of 2 modules in a row. Overall, the battery pack comprises 72 cells. The production volume is set at 25,000 packs per year.

The LFP and NMC 622 battery packs were simulated using the default values found in the BatPaC 5.0 model (high-energy configuration) and updating the costs of the main components according to the current prices.[9] The Zn||MnO_2 battery was simulated using the voltage vs. specific capacity discharge curve of Figure 2.a from the work of Pan et al. as input for the OCV points.[10] As remarked in the main text, we took care of eliminating the costs associated to the dry room and the negative electrode production for the rechargeable zinc battery. In fact, the aqueous electrolyte removes the need of a dry environment for the materials and battery processing. Additionally, as the negative electrode consists of a zinc metal foil, there are no coating and drying processes required to produce it. All the main parameters of the simulations are reported in **Table S2**, and the main results in **Table S3**.

Table S2 – Main parameters used in the simulation of the battery packs

Parameter	Zn||MnO_2 battery	Graphite||LFP battery	Graphite||NMC_{622} battery
Positive electrode active material specific capacity [$mAh \cdot g^{-1}$]	300	157	187
Positive electrode active material density [$g \cdot cm^{-3}$]	5.03	3.45	4.65
Positive electrode composition (AM:CC:B, weight fractions)	94:3:3	93:3:4	94:3:3
Positive electrode active material cost [$\$ \cdot kg^{-1}$]	2.30	7.00	25.00
Positive electrode current collector thickness [µm]	20 (Stainless steel)	15 (Aluminium)	15 (Aluminium)
Positive electrode current collector cost [$\$ \cdot m^{-2}$]	0.8	0.2	0.2
Negative electrode active material specific capacity [$mAh \cdot g^{-1}$]	820	360	360
Negative electrode active material density [$g \cdot cm^{-3}$]	7.14	2.26	2.26
Negative electrode composition (AM:CC:B, weight fractions)	100:0:0	96:2:2	96:2:2
Negative electrode active material cost [$\$ \cdot kg^{-1}$]	2.50	8.00	8.00
Negative electrode current collector thickness [µm]	20 (Stainless steel)	10 (Copper)	10 (Copper)
Negative electrode current collector cost [$\$ \cdot m^{-2}$]	0.8	1.1	1.1
Electrolyte density [$g \cdot cm^{-3}$]	1.19	1.21	1.21
Electrolyte cost [$\$ \cdot L^{-1}$]	0.5	3.5	4.5
N/P ratio[a]	1.1	1.1	1.1

[a] Ratio between the capacity of the anode and the capacity of the cathode

Table S3 – Main results of the simulation of the battery packs

Parameter	Zn||MnO_2 battery	Graphite||LFP battery	Graphite||NMC_{622} battery
Positive electrode active material cost [$\$\cdot pack^{-1}$]	84	178	478
Negative electrode active material cost [$\$\cdot pack^{-1}$]	36	101	92
Electrodes preparation cost [$\$\cdot pack^{-1}$]	78	60	46
Positive electrode current collector cost [$\$\cdot pack^{-1}$]	54	10	6
Negative electrode current collector cost [$\$\cdot pack^{-1}$]	56	61	37
Separators cost [$\$\cdot pack^{-1}$]	13	11	6
Electrolyte cost [$\$\cdot pack^{-1}$]	2	19	17
Battery system total energy [kWh]	11.5	11.5	11.5
Battery system rated power [kW]	7	7	7
Battery system capacity, [Ah]	127	48	44
Battery system nominal operating voltage [V]	90	238	264
Positive electrode thickness [µm]	98	120	120
Negative electrode thickness [µm]	18	83	129
Cell volume [L]	0.35	0.39	0.29
Cell mass [kg]	0.84	0.70	0.54
Cell capacity [Ah]	127	48	44
Cell specific energy [$Wh\cdot kg^{-1}$]	189	227	297
Cell energy density [$Wh\cdot L^{-1}$]	451	410	549
Cell cost [$\$\cdot kWh^{-1}$]	72	79	96

Bibliography

1. Zinc sulphate. https://www.alibaba.com/showroom/zinc-sulphate.html (Accessed on 26.11.2023).
2. Zinc chloride. https://www.alibaba.com/showroom/zncl2.html (Accessed on 26.11.2023).
3. Zinc acetate . https://www.alibaba.com/showroom/zinc-acetate-price.html (Accessed on 26.11.2023).
4. Perry's chemical engineers' handbook. *Choice (Middletown)* **35**, 35-3079-35–3079 (1998).
5. Alumeco. Metal price news - November 2023. https://www.alumeco-service.de/en/knowledge-technique/metal-price-news-2023/metal-price-news-november-2023/?s=0 (Accessed on 26.11.2023).
6. China Copper Spot price. https://www.metal.com/Copper (Accessed on 26.11.2023).
7. China Aluminum Spot price. https://www.metal.com/Aluminum (Accessed on 26.11.2023).
8. Nelson, P. A., Ahmed, S., Gallagher, K. G. & Dees, D. W. *Modeling the performance and cost of lithium-ion batteries for electric-drive vehicles, third edition*. https://www.osti.gov/biblio/1503280 (2019) doi:10.2172/1503280.
9. Lithium-ion battery components price. https://www.metal.com/Ternary-precursor-material (Accessed on 26.11.2023).
10. Pan, H. *et al.* Reversible aqueous zinc/manganese oxide energy storage from conversion reactions. *Nat. Energy* **1**, 16039 (2016).
